# The Treatment of Ulcers with Saturated Solution Chlorate Potassium

**Published:** 1878-12

**Authors:** T. M. Rochester

**Affiliations:** Brooklyn, N. Y.


### The Treatment of Ulcers with a Saturated Solution of Chlorate of Potassium.

BY T. M. ROCHESTER, M. D., BROOKLYN, N. Y.

I wish to call attention to the treatment of ulcers, ulcerations, and suppurating wounds and sores in general, by the use of a saturated solution of chlorate of potassium. I have said suppurating sores in general, and so it will be found beneficial in all of these; but it is especially useful in that particular class of old, unhealthy, indolent ulcers which are one of the opprobria of our art. Let me describe briefly my plan of treatment in one of these cases.

Suppose, for example, a patient comes with an old, indolent ulcer on the leg, which is the most frequent situation. The sore is foul, unhealthy in appearance, and ill-smelling. The limb is usually much swollen, granulation is at a stand-still, the ulcer may even be phagedenic, and the discharge is thin, sanious and offensive. As a rule, the patient has the sore covered with some salve or ointment, and the limb is either bandaged tightly or strapped with adhesive plaster.

In a case of this kind I immediately remove all bandages and constrictions, and direct that they shall not be again applied. The ulcer is then carefully and gently washed with warm water, after which it is ordered to be gently douched five or six times a day with a saturated solution of chlorate of potassium; my usual prescription is: Take of potassii chloratis, 2 drachms; aquæ puræ, one pint. The best method of doing this is to fill a sponge with the solution, and then slowly press it out, holding it within an inch or two of the sore. After the ulcer has been quite freely douched, a thin layer of the unguentum zinci oxidi should be spread around the edges. It is then to be completely covered with a piece of oiled lint, kept in place by a bandage applied as loosely as possible. The douching should be repeated, at first, five or six times daily; the zinc ointment need not be used more than twice in the twenty-four hours. At the end of two or three days the ulcer will begin to assume a healthy appearance, and commence to granulate nicely. When this improvement is noticed, the number of douchings should be decreased—otherwise the granulations will become pale and flabby. As the sore fills up and begins to heal over around the edges, the zinc ointment should be increased, so as to cover the new skin. This serves to protect and strengthen it. So, too, it is well to continue its use for a few days after the ulcer has completely skinned over.

Such, in brief, is my mode of dealing with these cases; and it is with the keenest satisfaction that I have watched case after case of unhealthy, indolent, varicose or specific ulcerations, which have defied one treatment after another, and worn out the patience of physician and patient alike, take on a healthy appearance after two or three days of this treatment, and thence proceed rapidly to a complete recovery. During the past fifteen months I have seen used and treated by this plan between ninety and one hundred cases, and in no single instance have failed to see immediate and continued improvement, and, when they could be kept track of, completely healing in one to six weeks.

This number of cases embraces not only the ordinary indolent ulcers, but also syphilitic, varicose, and one case which I believe was carcinomatous in its character.

Several of my cases had had scraping and skin-grafting tried in vain. Where they were syphilitic, constitutional treatment had been and was used alone without effect; but rapid healing immediately took place when the douching with the saturated solution of chlorate of potassium was combined with it.

There is another point that is worth mentioning, and that is, that this douching is entirely painless after a few applications. It may smart a little the first or second time, but even then it is so slight as not to be taken into consideration. Now to mention a few cases, as it would only be tedious to give them all. The following are fair samples of the whole, as they are taken entirely at random and not selected at all, with the exception of the first, which is a very marked example of the efficacy of this treatment.

CASE.—C. K., male, aged 29, was sent to the hospital to have leg amputated at the middle third of the thigh, as a last resort. Had an ulcer three inches by one and a half on the external, lateral aspect of the leg. The limb was swollen to twice the natural size, and gangrenous in appearance as far as the lower third of the thigh. The discharge from the ulcer was thin, sanious and very fetid. There was no history of syphilis, either congenital or acquired. Had been getting gradually worse for over a year, although he had tried almost everything, and seen a number of physicians in regard to it. He walked with great pain and difficulty. The solution of chlorate of potassium was used, and by the third day a decided improvement was noticed. The swelling of the leg had decreased, and the ulcer began to granulate nicely. At this time he had an attack of delirium tremens, which lasted four or five days; but notwithstanding this shock to the nervous system, the leg continued to improve *visibly* from day to day. At the end of four weeks the limb was of the normal size and appearance, the ulcer healthy, and about as large as a three cent piece. The patient could walk without any inconvenience, and insisted on leaving the hospital, to fill the position of organist in a Catholic church, although strongly advised not to. He returned after a week or two with the leg slightly swollen, and the ulcer about the size of a silver quarter. The treatment was resumed, and he was discharged cured in ten days.

CASE.—A. G., female, aged 30. Syphilitic. Large deep ulcer on the posterior aspect of leg. Discharge excessively foul and unhealthy. Had tried specific treatment alone in vain. Leg healed nicely in about five weeks, under the combined use of constitutional and the chlorate of potassium treatment.

CASE.—M. W., female, aged 66, syphilitic. An extremely large and deep ulcer, covering nearly the whole of the posterior and external lateral aspects of the leg. Discharge excessively fetid. Rapid and continued improvement for several weeks, under the combined constitutional and chlorate of potassium treatment. Lost sight of before complete healing took place.

CASE.—J. H., male, aged 66. Several small but troublesome ulcers along the crest of the tibia. Had been troubled for months. Probably due to a previous injury of the knee-joint. Healed completely in ten days by this treatment.

CASE.—P. M., male, aged 38. Varicose ulcer of leg. Had been troubled for over a year. Healed completely in one week.

Case.—O. H., male, aged 46. Small indolent ulcer of the leg of many months' standing. Healed in one week.

Case.—C. K., female, aged 40. Large indolent ulcer of the leg of over a year's standing. Rapid and continued improvement for two weeks. Was then lost sight of.

Case.—P. N., male, aged 38. Troubled with a large varicose ulcer for several years. Healed completely in three weeks.

Case.—S. T., female, aged 10. Simple but unhealthy-looking ulcer of several weeks' duration. Healed in one week.

Case.—M. C., female, aged 40. Varicose ulcer of several years' standing. Healed in four weeks.

Case.—F. F., male, aged 19. Two large ragged, deep and unhealthy-looking sores on the right leg, and a similar one on the left. Discharge excessively fetid. Looked as though gangrene had set in. Healed completely in three weeks.

Case.—M. M., female, aged 40. Large indolent ulcer of the leg of several years' standing. Healed in five weeks.

Case.—L. C., male, aged 41. Troubled for years with an indolent ulcer of the leg. Had tried almost everything—amongst other means, skin-grafting—but without benefit. Healed in two weeks.

And so I might go on, and enumerate case after case; but I think the above are sufficient the prove the efficacy of this plan of treatment. I wish, however, to quote one case, as showing the advantage in the use of the saturated solution of chlorate of potassium in recent injuries when the suppuration is profuse;

Case.—H. B., male, aged 28, of fine muscular development. Received a penetrating lacerated wound of the arm, which severed the brachial artery. The hemorrhage was controlled by acu-pressure both above and below the wound. On the removal of the needles a large slough came away, and the suppuration was excessive and very exhausting. The solution of chlorate of potassium was used, the excessive flow of pus was checked, the fetor arising from it allayed, and the patient rapidly convalesced.

One word as regards the mode of operation of chlorate of potash thus used. I do not know that I am right, but my theory is as follows:

1st. Its astringent properties cause it to repress exuberant granulations, or to stimulate their growth when they are absent or ill-formed.

2d. As an antiphlogistic it moderates the inflammatory action.

3d. And this I regard as the most important: as an antiseptic it destroys and prevents the growth of bacteria.

This threefold action, I believe, meets all the indications for the local treatment of this troublesome class of afflictions. In conclusion, let me say that this use of chlorate of potassium is not my own discovery, but was suggested to me while *interne* at the Monroe County Hospital, by the attending physician, Dr. Azel Backus, of Rochester, who has used this treatment successfully in his practice for years.

